# Staffing and Antipsychotic Medication Use in Nursing Homes and Neighborhood Deprivation

**DOI:** 10.1001/jamanetworkopen.2024.8322

**Published:** 2024-04-24

**Authors:** Jasmine L. Travers, Erinn M. Hade, Steven Friedman, Aasha Raval, Kimberly Hadson, Jason R. Falvey

**Affiliations:** 1 New York University Rory Meyers College of Nursing, New York; 2 New York University Grossman School of Medicine, Department of Population Health, New York; 3 University of Maryland School of Medicine, Department of Physical Therapy and Rehabilitation Science, Department of Epidemiology and Public Health, Baltimore

## Abstract

**Importance:**

Inappropriate use of antipsychotic medications in nursing homes is a growing public health concern. Residents exposed to higher levels of socioeconomic deprivation in the area around a nursing home may be currently exposed, or have a long history of exposure, to more noise pollution, higher crime rates, and have less opportunities to safely go outside the facility, which may contribute to psychological stress and increased risk of receiving antipsychotic medications inappropriately. However, it is unclear whether neighborhood deprivation is associated with use of inappropriate antipsychotic medications and whether this outcome is different by facility staffing levels.

**Objective:**

To evaluate whether reported inappropriate antipsychotic medication use differs in severely and less severely deprived neighborhoods, and whether these differences are modified by higher levels of total nurse staffing.

**Design, Setting, and Participants:**

This was a cross-sectional analysis of a national sample of nursing homes that linked across 3 national large-scale data sets for the year 2019. Analyses were conducted between April and June 2023.

**Exposure:**

Neighborhood deprivation status (severe vs less severe) and total staffing hours (registered nurse, licensed practical nurse, certified nursing assistant).

**Main Outcome and Measures:**

This study estimated the association between neighborhood deprivation and the percentage of long-stay residents who received an antipsychotic medication inappropriately in the nursing home at least once in the past week and how this varied by nursing home staffing through generalized estimating equations. Analyses were conducted on the facility level and adjusted for state fixed effects.

**Results:**

This study included 10 966 nursing homes (1867 [17.0%] in severely deprived neighborhoods and 9099 [83.0%] in less deprived neighborhoods). Unadjusted inappropriate antipsychotic medication use was greater in nursing homes located in severely deprived neighborhoods (mean [SD], 15.9% [10.7%] of residents) than in those in less deprived neighborhoods (mean [SD], 14.2% [8.8%] of residents). In adjusted models, inappropriate antipsychotic medication use was higher in severely deprived neighborhoods vs less deprived neighborhoods (19.2% vs 17.1%; adjusted mean difference, 2.0 [95% CI, 0.35 to 3.71] percentage points) in nursing homes that fell below critical levels of staffing (less than 3 hours of nurse staffing per resident-day).

**Conclusions and Relevance:**

These findings suggest that levels of staffing modify disparities seen in inappropriate antipsychotic medication use among nursing homes located in severely deprived neighborhoods compared with nursing homes in less deprived neighborhoods. These findings may have important implications for improving staffing in more severely deprived neighborhoods.

## Key Points

**Question:**

Is there a difference in reported inappropriate antipsychotic medication use between severely and less severely deprived neighborhoods, and is this difference modified by greater total nurse staffing hours?

**Findings:**

In this cross-sectional study of 10 966 nursing homes, nursing homes that fell below critical levels of staffing (ie, less than 3 hours of nurse staffing per resident-day), were associated with higher inappropriate antipsychotic medication use among nursing homes in severely deprived neighborhoods (19.2%) compared with nursing homes in less deprived neighborhoods (17.1%).

**Meaning:**

These findings suggest that addressing staffing deficiencies in nursing homes, particularly those located in severely deprived neighborhoods, is crucial in mitigating inappropriate antipsychotic medication use.

## Teaser

This cross-sectional study evaluates whether reported inappropriate antipsychotic medication use differs among nursing homes in neighborhoods that are severely deprived or less severely deprived and whether these differences are associated with level of total nurse staffing.

## Introduction

Inappropriate use of antipsychotic medications in nursing homes is a growing public health concern, with recent evidence suggesting use of these medications among older adults is associated with serious events including cognitive impairment, falls, and higher mortality rates.^1^ The US Centers for Medicare & Medicaid Services (CMS) tracks facility-level use of antipsychotic medications for conditions other than approved disease states, such as schizophrenia, Huntington disease, and Tourette syndrome; and while not tracked, but considered an approved disease state, a predetermined or long-standing history of mental illness; and in rare circumstances, for people living with dementia.^2^ CMS publicly reports a corresponding quality measure for each facility on Nursing Home Care Compare—this measure reflects the rate of antipsychotic medication use among long-stay residents without a qualifying clinical indication.

Prior work suggests that adults living in socioeconomically deprived neighborhoods, which have higher rates of crime,^3^ less available green space,^4^ poorer quality built environments (eg, sidewalks),^5^ and higher noise pollution^6^ may be more vulnerable to both psychological and cognitive declines.^7^ Residents of nursing homes located in these areas are not immune to these neighborhood concerns, which may impact sleep, social functioning, and exposure to green space due to limited opportunities for outings outside the nursing home, and ultimately contribute to mental and physical health stressors^8^ that may manifest with behavioral symptoms or agitation. Together, these factors may lead to a higher likelihood of antipsychotic medication use.

Higher antipsychotic medication use is also observed in facilities with lower rates of staffing, especially among registered nurses.^9^ Prior work has shown facilities in deprived neighborhoods have lower rates of registered nurse staffing^10^—added vulnerability for residents in these nursing homes that may further magnify the risk for antipsychotic medication use inappropriately. Understanding this multilevel vulnerability would inform potential policy solutions to support, instead of penalizing, facilities in disinvested communities. Thus, the purpose of this study was to evaluate whether reported inappropriate antipsychotic medication use is associated with neighborhood deprivation, and whether these associations are modified by higher levels of total nurse staffing (as defined by hours from registered nurses [RNs], licensed practical nurses [LPNs], and certified nursing assistants [CNAs] inclusive of administrative hours).

## Methods

### Design and Datasets

This was a cross-sectional analysis linking multiple national large-scale data sets from 2019. Data sources were all publicly available and included Nursing Home Care Compare for resident outcomes and facility characteristics (fourth quarter means), payroll-based journal (PBJ) for staffing hours (average over the full year), and the Area Deprivation Index (ADI) for neighborhood deprivation. No nursing homes were excluded if they linked together across the 3 data sources. This study met predetermined criteria for institutional review board and informed consent exemption according to the Common Rule due to the use of deidentified data. This study followed the Strengthening the Reporting of Observational Studies in Epidemiology (STROBE) reporting guideline.

### Neighborhood Deprivation

Each facility address from Nursing Home Care Compare was geocoded to the census-block group level, and then merged with the ADI (independent variable of interest) using commercially available software to obtain a composite national ranking of socioeconomic deprivation at the facility level (1 to 100, higher scores indicating more deprivation). Consistent with the threshold theory of poverty, which states that neighborhood poverty will impact older adults most significantly at the extremes, neighborhood deprivation was categorized as severe (ADI of 85 or higher) or not severe (ADI of less than 85). An 85th percentile cut point in national data has been used previously, which is consistent with this study.^11^

#### Facility Nurse Staffing

Facility nurse staffing was measured as the total nursing hours per resident per day, combining hours recorded in PBJ data for RNs, LPNs, and CNAs. This was coded as a categorical variable of more than 4.1 hours per resident per day (recommended),^12^ 3 to 4.1 hours per resident per day (marginal),^13^ and less than 3 hours per resident per day (substandard),^13^ based on the proposed minimum threshold for total staffing hours of 4.1 hours per resident per day.^12^

#### Inappropriate Antipsychotic Medication Use

The outcome of interest was inappropriate antipsychotic medication use, measured as a percentage on the facility level in Nursing Home Care Compare. We used the 2019 long-stay variable which reflects the percentage of long-stay residents who received an antipsychotic medication in the nursing home at least once in the past week without a diagnosis of schizophrenia, Huntington disease, or Tourette syndrome.

#### Resident and Facility Characteristics

Characteristics of the resident were aggregated on the facility level and consisted of several quality measures from Nursing Home Care Compare, including depression measured as a percentage of residents experiencing the condition on the facility level. Facility characteristics included bed size, ownership status, acuity index, and rurality.

### Statistical Analysis

All data sets were merged or linked by the nursing home identification number after the ADI data were geocoded to Nursing Home Care Compare. We examined descriptive aggregated resident and facility characteristics of the study sample to compare characteristics of facilities located in severely deprived neighborhoods with those in less deprived neighborhoods. We estimated the association between neighborhood deprivation and the percentage of antipsychotic medication use in nursing home facilities, and stratification of this effect by facility staffing through generalized linear models estimated by generalized estimating equations (GEE). The stratified association of neighborhood deprivation by facility staffing was tested through the inclusion of an interaction term in our models. Our goal was to estimate the association for the population mean association of neighborhood deprivation with inappropriate antipsychotic medication use, with stratification by facility staffing; however, facilities and neighborhoods are unlikely to be independent in this association across geography. Therefore, we used GEE with robust sandwich-type standard errors to accommodate this potential clustering by county, with additional adjustment for state as a fixed effect. The reported analyses are for the complete cases and missing data considered missing at random. Those nursing homes that did not link had higher CNA and RN hours, fewer beds, and lower percentage of nursing homes that were for-profit compared with nursing homes that did match, and they had similar inappropriate antipsychotic medication use as nursing homes in less deprived neighborhoods (eTable 1 in Supplement 1). Variability was assessed with 2-sided 95% CIs, and all analyses were conducted in SAS, version 9.4 (SAS Institute) between April and June 2023.

## Results

Our study included the 10 966 nursing homes (1867 [17.0%] in severely deprived neighborhoods and 9099 [83.0%] in less deprived neighborhoods) that linked across all data sets (3360 were missing). Severely deprived nursing homes were more likely to be for-profit, in rural areas, and have fewer beds (Table). We observed no meaningful differences in quality care indicators that might be associated with antipsychotic medication use between less and severely deprived nursing homes, including depression (Table and eTable 2 in Supplement 1).

**Table.** Descriptive Statistics of Nursing Homes Located in Severely Deprived Neighborhoods and Nursing Homes Located in Less Deprived Neighborhoods

Characteristics	Nursing homes, No. (%)		
	All (N = 10 966)	Less deprived (n = 9099)	Severely deprived (n = 1867)
Staffing hours per MDS census			
Certified nursing assistant, mean (SD)	2.1 (0.5)	2.2 (0.5)	2.1 (0.5)
Greater than 2.81 h per MDS	906 (8.3)	811 (9.0)	95 (5.1)
Licensed practical nurse, mean (SD)	0.9 (0.3)	0.8 (0.3)	0.9 (0.3)
Greater than 0.54 h per MDS	9322 (85.4)	7673 (84.7)	1649 (88.9)
Registered nurse, mean (SD)	0.6 (0.4)	0.7 (0.4)	0.5 (0.3)
Greater than 0.75 h per MDS	3070 (28.1)	2793 (30.8)	277 (14.9)
Total nursing staff hours			
Mean (SD)	3.6 (0.8)	3.7 (0.8)	3.4 (0.7)
>4.1 h per MDS	2334 (21.4)	2091 (23.1)	243 (13.1)
3-4.1 h per MDS	6658 (61.0)	5483 (60.5)	1175 (63.4)
<3 h per MDS	1922 (17.6)	1485 (16.4)	437 (23.6)
Deficiencies			
None	9486 (86.5)	7925 (87.1)	1561 (83.6)
≥1	1480 (13.5)	1174 (12.9)	306 (16.4)
Nursing home resident quality care outcomes, mean (SD)			
ADL need increased	14.9 (6.1)	14.7 (6.1)	15.6 (6.1)
Pressure ulcer	7.4 (4.2)	7.3 (4.2)	7.7 (4.4)
Urinary tract infection	2.7 (2.7)	2.7 (2.7)	2.8 (2.7)
Depressed	5 (10.8)	5 (10.9)	5.2 (10.7)
Restrained	0.2 (1.3)	0.2 (1.3)	0.3 (1.2)
Falls	3.4 (2.3)	3.4 (2.3)	3.5 (2.3)
Flu vaccination	95.7 (6.7)	95.7 (6.7)	95.7 (6.9)
Pneumonia vaccination	93.4 (12.0)	93.4 (11.9)	93.2 (12.7)
Antipsychotic prescription	14.5 (9.2)	14.2 (8.8)	15.9 (10.7)
Worsened mobility	18 (7.3)	18 (7.3)	17.8 (7.5)
Antianxiety	20.4 (9.9)	19.9 (9.7)	23 (10.6)
Lose bladder or bowel control	49.3 (17.2)	50.2 (17.1)	45 (17.3)
Lose too much weight	5.5 (3.2)	5.4 (3.2)	5.8 (3.5)
Catheterized	1.9 (2.0)	1.9 (2.0)	2 (1.8)
Alzheimer unit, No. (%)	1567 (14.3)	1317 (14.5)	250 (13.4)
Chain ownership, No. (%)	6696 (61.1)	5523 (60.7)	1173 (62.8)
Nursing home characteristics			
Certified bed size, mean (SD)	109 (56.1)	110.4 (57.7)	102 (46.9)
Organization ownership status			
For profit	7846 (71.5)	6445 (70.8)	1401 (75.0)
Government	565 (5.2)	434 (4.8)	131 (7.0)
Nonprofit	2555 (23.3)	2220 (24.4)	335 (17.9)
Acuity index, mean (SD)	12.1 (1.7)	12.1 (1.7)	12 (1.6)
Rural code			
Metropolitan	7898 (72.0)	6976 (76.7)	922 (49.4)
Nonmetropolitan			
Adjacent to metropolitan area	1954 (17.8)	1380 (15.2)	574 (30.7)
Not adjacent to metropolitan area	1114 (10.2)	743 (8.2)	371 (19.9)

Abbreviation: MDS, Minimum Data Set.

Unadjusted estimates of antipsychotic medication use were greater in nursing homes located in severely deprived neighborhoods (mean [SD], 15.9% [10.7%] of residents) than in those in less deprived neighborhoods (mean [SD], 14.2% [8.8%] of residents), but these differences were modified by nursing home staffing levels (Figure). Adjusted for state, we observed that in nursing homes that fell below 3 hours of nurse staffing per resident-day, antipsychotic medication use was higher in severely deprived neighborhoods vs less deprived neighborhoods (19.2% vs 17.1%; adjusted mean difference, 2.0 [95% CI, 0.35%-3.71%] percentage points). Similar, albeit weaker associations were observed for facilities staffed between 3 and 4.1 hours per resident-day (15.0% antipsychotic medication use rates in severely deprived neighborhoods vs 13.9% in less deprived neighborhoods; adjusted mean difference, 1.17 [95% CI, 0.60%-1.73%] percentage points). Neighborhood deprivation was not significantly associated with higher antipsychotic medication use in facilities that met or exceeded the proposed threshold for staffing levels.

**Figure.**
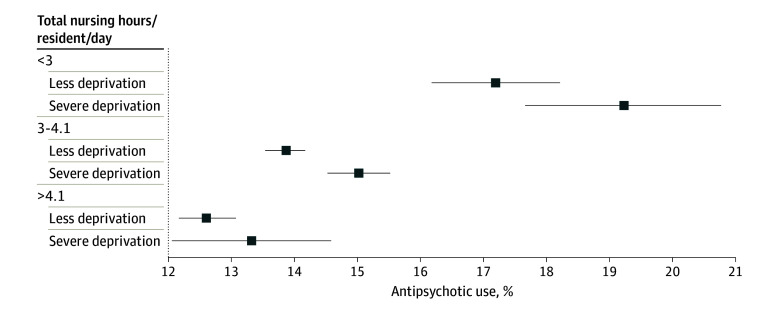
Percentage of Antipsychotic Medication Use by Neighborhood Disadvantage and Staffing Level Data were adjusted for fixed effects of state and clustering of facilities by county. The recommended total nurse staffing hours was more than 4.1, marginal total nursing staffing hours was from 3.0 to 4.1 hours, and substandard total nursing staffing hours was less than 3. Boxes indicate mean and error bars indicate 95% CIs.

## Discussion

Our findings suggest that neighborhood-level socioeconomic deprivation was associated with greater use of inappropriate antipsychotic medication use in nursing homes, and this association was greatest in facilities with the lowest nursing staffing rates. Our data indicated that in a 100-bed nursing home with nurse staffing levels less than 3 hours per resident day, an estimated 2 additional residents would receive antipsychotic medications if that facility was located in socioeconomically deprived neighborhoods vs in less deprived neighborhoods. Inappropriate antipsychotic medication use is associated with increased risk of cognitive and mobility decline and elevated mortality risk among residents.^1^ The association of neighborhood deprivation with inappropriate antipsychotic medication varied by facility staffing levels, with the largest impact in those with the least staffing, thus providing support for federally mandated minimum nursing home staffing levels.^14^

Providing adequate staffing in nursing homes, particularly those in deprived neighborhoods, may be critical to reducing inappropriate antipsychotic medication use. Evidence suggested that staffing was important to this and other aspects of quality of care, but maintaining a well-staffed and adequately trained facility in neighborhoods that are severely deprived may be more challenging.^15,16^

While minimum staffing standards have been requested by both the Biden-Harris administration and the National Academies of Medicine,^17,18^ there is considerable tension within the industry regarding the implementation of these recommendations while acknowledging the unique needs of each facility.^19^ Initiatives, such as the CMS National Partnership to Improve Dementia Care in Nursing Homes, which promotes person-centered, nonpharmacological approaches to managing dementia-related behaviors,^20^ may be more challenging in neighborhoods that are more deprived. Higher staff turnover and fiscal stress suggests lower available resources for effectively implementing these evidence-based interventions.^21^ Lack of resources (eg, providing time outdoors given neighborhood safety concerns or poorer quality-built environments) may prevent consistently implemented nonpharmacological management strategies. Staff working in nursing homes in deprived areas may also be more risk averse, have more environmental safety concerns, and have a lower capacity to develop individualized care plans. Our work in this study, leveraging national data linked with census-block level geographic metrics of poverty, identified how neighborhood context might impact inappropriate antipsychotic medication use and called for more focused attention on improving staffing efforts in these neighborhoods. For example, instead of penalizing nursing homes located in deprived neighborhoods and with poorer quality outcomes, such as increased antipsychotic medication use, state and federal entities could focus on targeting funds at the zip code level to better support staffing initiatives in these areas. This has been done for other health care settings located in deprived neighborhoods, such as home health care and federally qualified health centers. Moreover, such facilities may need more than staffing support (ie, attention to factors inherent to the neighborhood) to reduce inappropriate antipsychotic medication use.

### Limitations

This study has limitations. The associations drawn in our study are not causal, and the potential for unmeasured confounding exists. For example, in addition to the 3 conditions already excluded from the inappropriate antipsychotic medication use variable, antipsychotic medications can also be used for people with a predetermined or long-standing history of mental illness and, in rare circumstances, for people living with dementia who were not already excluded from the Nursing Home Care Compare antipsychotic medication use variable. Because of data limitations, we are unable to assess the difference in these 2 conditions across the exposure, nor control for them. Although we accounted for state fixed effects, it is possible that there could have been facility-level policies focused on the administration of antipsychotic medications that were existent in some nursing homes and not others not associated with neighborhood deprivation that we were not able to account for. Lastly, as 3360 nursing homes did not link across all data sets, there is a possibility for potential selection bias.

## Conclusions

In this study, the associations between neighborhood-level socioeconomic deprivation and facility-level inappropriate antipsychotic medication use in nursing homes were strongest in nursing homes with the lowest staffing levels. Facilities in lower income areas may need more tailored interventions to address staffing gaps; future research should support helping socioeconomically deprived facilities meet staffing standards.

## Notes

Supplement 1. **eTable 1.** Missing Nursing Homes That Did Not Link Across Datasets **eTable 2.** Differences in Resident Quality Care Outcomes Between Nursing Homes in Severely Deprived Neighborhoods vs Nursing Homes in Less Deprived Neighborhoods

Supplement 2.
Data Sharing Statement

## References

[1] Kirkham J, Sherman C, Velkers C, . Antipsychotic Use in Dementia. Can J Psychiatry. 2017;62(3):170-181. doi:10.1177/0706743716673321 28212496 PMC5317021

[2] National Partnership to Improve Dementia Care in Nursing Homes. Antipsychotic Medication Use Data Report. Centers for Medicare & Medicaid Services (CMS). Accessed March 12, 2023. https://www.cms.gov/files/document/antipsychotic-medication-use-data-report-2021q4-updated-07292022.pdf

[3] Henry OS, Batchu S, Lachant J, . Disadvantaged neighborhoods continue to bear the burden of gun violence. J Surg Res. 2024;293:396-402. doi:10.1016/j.jss.2023.09.002 37806227

[4] de Keijzer C, Bauwelinck M, Dadvand P. Long-Term exposure to residential greenspace and healthy aging: a systematic review. Curr Environ Health Rep. 2020;7(1):65-88. doi:10.1007/s40572-020-00264-7 31981136

[5] Kapsalis E, Jaeger N, Hale J. Disabled-by-design: effects of inaccessible urban public spaces on users of mobility assistive devices—a systematic review. Disabil Rehabil Assist Technol. 2022;2022:1-19. doi:10.1080/17483107.2022.2111723 35984675

[6] Casey JA, Morello-Frosch R, Mennitt DJ, Fristrup K, Ogburn EL, James P. Race/ethnicity, socioeconomic status, residential segregation, and spatial variation in noise exposure in the contiguous United States. Environ Health Perspect. 2017;125(7):077017. doi:10.1289/EHP898 28749369 PMC5744659

[7] Meng L, Zhang Y, Zhang S, . Chronic noise exposure and risk of dementia: a systematic review and dose-response meta-analysis. Frontiers Public Health. 2022;10:832881. doi:10.3389/fpubh.2022.832881 PMC9251202 35795699

[8] Choi YJ, Matz-Costa C. Perceived neighborhood safety, social cohesion, and psychological health of older adults. Gerontologist. 2017:196-206. doi:10.1093/geront/gnw187 28082279

[9] Chappell V, Kirkham J, Seitz DP. Association between long-term care facility staffing levels and antipsychotic use in us long-term care facilities. J Am Med Dir Assoc. 2022;23(11):1787-1792.e1. doi:10.1016/j.jamda.2022.06.029 35926573

[10] Falvey JR, Hade EM, Friedman S, . Severe neighborhood deprivation and nursing home staffing in the United States. J Am Geriatr Soc. 2023;71(3):711-719. doi:10.1111/jgs.17990 36929467 PMC10023834

[11] Kind AJ, Jencks S, Brock J, . Neighborhood socioeconomic disadvantage and 30-day rehospitalization: a retrospective cohort study. Ann Intern Med. 2014;161(11):765-774. doi:10.7326/M13-2946 25437404 PMC4251560

[12] Report: increasing nursing home staffing minimums estimated at $10 billion annually. American Health Care Association. Accessed February 7, 2024. https://www.ahcancal.org/News-and-Communications/Press-Releases/Pages/Report-Increasing-Nursing-Home-Staffing-Minimums-Estimated-at-$10-Billion-Annually.aspx

[13] Burns A, Chidambaram P, Neuman T, Rudowitz R. What share of nursing facilities might meet proposed new requirements for nursing staff hours? KFF. Accessed February 7, 2024. https://www.kff.org/medicaid/issue-brief/what-share-of-nursing-facilities-might-meet-proposed-new-requirements-for-nursing-staff-hours/

[14] Karikari-Martin P. Centers for Medicare & Medicaid Services staffing study to inform minimum staffing requirements for nursing homes. Centers for Medicare & Medicaid Services. Accessed March 12, 2024. https://www.cms.gov/blog/centers-medicare-medicaid-services-staffing-study-inform-minimum-staffing-requirements-nursing-homes

[15] Stone RI, Travers JL, Falvey JR. Neighborhood deprivation and nursing home staffing: lessons for policy and practice. LTSS Center. Accessed February 7, 2024. https://ltsscenter.org/reports/Neighborhood_Deprivation_and_Nursing_Home_Staffing.pdf

[16] Fashaw-Walters SA, McCreedy E, Bynum JPW, Thomas KS, Shireman TI. Disproportionate increases in schizophrenia diagnoses among Black nursing home residents with ADRD. J Am Geriatr Soc. 2021;69(12):3623-3630. doi:10.1111/jgs.17464 34590709 PMC8648979

[17] Fact sheet: Biden-Harris administration announces new steps to improve quality of nursing homes. The White House. Accessed February 7, 2024. https://www.whitehouse.gov/briefing-room/statements-releases/2022/10/21/fact-sheet-biden-harris-administration-announces-new-steps-to-improve-quality-of-nursing-homes/

[18] The national imperative to improve nursing home quality: honoring our commitment to residents, families, and staff. National Academies Press. Accessed March 12, 2024. https://nap.nationalacademies.org/read/26526/chapter/1 36198022

[19] Should nursing homes come with an antipsychotic drug warning? Levin and Perconti. Accessed March 12, 2024. https://www.levinperconti.com/blog/should-nursing-homes-come-with-an-antipsychotic-drug-warning

[20] National partnership to improve dementia care in nursing homes. Centers of Medicare & Medicaid. Accessed February 7, 2024. https://www.cms.gov/medicare/provider-enrollment-and-certification/surveycertificationgeninfo/national-partnership-to-improve-dementia-care-in-nursing-homes

[21] Walsh KA, Dennehy R, Sinnott C, . Influences on decision-making regarding antipsychotic prescribing in nursing home residents with dementia: a systematic review and synthesis of qualitative evidence. J Am Med Dir Assoc. 2017;18(10):897.e1-897.e12. doi:10.1016/j.jamda.2017.06.032 28807433

