# Supplementary material for: Staffing and Antipsychotic Medication Use in Nursing Homes and Neighborhood Deprivation
Source: JAMA Netw Open. 2024 Apr 24;7(4):e248322. doi: 10.1001/jamanetworkopen.2024.8322 (PMC11043897; doi:10.1001/jamanetworkopen.2024.8322)
Supplement: Supplement 1. — eTable 1. Missing Nursing Homes That Did Not Link Across Datasets eTable 2. Differences in Resident Quality Care Outcomes Between Nursing Homes in Severely Deprived Neighborhoods vs Nursing Homes in Less Deprived Neighborhoods [file jamanetwopen-e248322-s001.pdf]

Supplemental Online Content

Travers JL, Hade EM, Friedman S, Raval A, Hadson K, Falvey JR. Staffing and antipsychotic medication use in nursing homes located in deprived neighborhoods. *JAMA Netw Open.* 2024;7(4):e248322. doi:10.1001/jamanetworkopen.2024.8322

eTable 1. Missing Nursing Homes That Did Not Link Across Datasets
eTable 2. Differences in Resident Quality Care Outcomes Between Nursing Homes in Severely Deprived Neighborhoods vs. Nursing Homes in Less Deprived Neighborhoods

This supplemental material has been provided by the authors to give readers additional information about their work.

© 2024 Travers JL et al. *JAMA Network Open.*

eTable 1. Missing Nursing Homes That Did Not Link Across Datasets

Mean (SD) or % (Count)	Unmatched (n-3360)
**Staffing Hours (per MDS census)**	
Certified Nursing Assistant	2.4 (0.6) missing=77
Licensed Practical Nurse	0.9 (00.5) missing=77
Registered Nurse	0.8 (0.8) missing-=77
Total Nursing staff	4.2 (1.2) missing=77
**Nursing Home Resident Quality Care Outcomes**	
ADL Need Increased	13.3 (6.6) missing=351
Pressure Ulcer	7.1 (4.8) missing=372
Urinary Tract Infection	2.7 (2.9) missing=307
Depressed	3.2 (8.3) missing=312
Restrained	0.4 (2.3) missing=302
Falls	3.1 (2.6) missing=302
Flu Vaccination	95.9 (7.4) missing=306
Pneumonia Vaccination	94.5 (11.1) missing=302
Antipsychotic Prescription	14.3 (11.7) missing=322
Worsened Mobility	16.5 (7.8) missing=393
Lose too much weight	5.2 (3.4) missing=317
Catheterized	2.2 (2.5) missing=312
Lose bladder/bowel control	45.9 (17.7) missing=473
Antianxiety	19.1 (10.6) missing=311
Alzheimer Unit	16.5% (54, 3033 missing)
Chain Ownership	45.9% (150, 3033 missing)
**Nursing Home Characteristics**	
Certified Bed Size	100.3 (71.9)
Organization Ownership Status	
For Profit	67.4% (2266)
Government	9.6% (324)
Non Profit	22.9% (770)
Acuity index	12.4 (1.3) missing=3033
Rural Code	
Metro	51% (597)
Non-metro – adjacent to metro	26.2 (307)
Non-metro – not adjacent to metro	22.8 (267)

© 2024 Travers JL et al. *JAMA Network Open.*

© 2024 Travers JL et al. *JAMA Network Open.*

eTable 2. Differences in Resident Quality Care Outcomes Between Nursing Homes in Severely Deprived Neighborhoods vs. Nursing Homes in Less Deprived Neighborhoods			
**Nursing Home Resident Quality Care Outcomes**	**Less Deprived (n=9099**	**Severely Deprived (n=1867)**	**Mean difference or Odds Ratio (95% CI)**
ADL Need Increased, mean (SD)	14.7 (6.1)	15.6 (6.1)	-0.82 (-1.12, -0.51)
Pressure Ulcer, mean (SD)	7.3 (4.2)	7.7 (4.4)	-0.42 (-0.64, -0.20)
Urinary Tract Infection, mean (SD)	2.7 (2.7)	2.8 (2.7)	-0.12 (-0.25, 0.02)
Depressed, mean (SD)	5 (10.9)	5.2 (10.7)	-0.17 (-0.71, 0.37)
Restrained, mean (SD)	0.2 (1.3)	0.3 (1.2)	-0.05 (-0.11, 0.01)
Falls, mean (SD)	3.4 (2.3)	3.5 (2.3)	-0.12 (-0.24, -0.01)
Flu Vaccination, mean (SD)	95.7 (6.7)	95.7 (6.9)	-0.08 (-0.42, 0.26)
Pneumonia Vaccination, mean (SD)	93.4 (11.9)	93.2 (12.7)	0.18 (-0.45, 0.80)
Antipsychotic Prescription, mean (SD)	14.2 (8.8)	15.9 (10.7)	-1.78 (-2.3, -1.26)
Worsened Mobility, mean (SD)	18 (7.3)	17.8 (7.5)	0.29 (-0.08, 0.65)
Antianxiety, mean (SD)	19.9 (9.7)	23 (10.6)	-3.11 (-3.63, -2.58)
Lose bladder/bowel control, mean (SD)	50.2 (17.1)	45 (17.3)	5.21 (4.35, 6.06)
Lose too much weight, mean (SD)	5.4 (3.2)	5.8 (3.5)	-0.36 (-0.54, -0.19)
Catheterized, mean (SD)	1.9 (2)	2 (1.8)	-0.04 (-0.13, 0.05)
Alzheimer Unit, n (%)	1317 (14.5)	250 (13.4)	0.91 (0.79, 1.06)
Chain Ownership, n (%)	5523 (60.7)	1173 (62.8)	1.09 (0.99, 1.21)

© 2024 Travers JL et al. *JAMA Network Open.*
