# Supplementary material for: Staffing and Antipsychotic Medication Use in Nursing Homes and Neighborhood Deprivation
Source: JAMA Netw Open. 2024 Apr 24;7(4):e248322. doi: 10.1001/jamanetworkopen.2024.8322 (PMC11043897; doi:10.1001/jamanetworkopen.2024.8322)
Supplement: Supplement 2. — Data Sharing Statement [file jamanetwopen-e248322-s002.pdf]

Data Sharing Statement

Travers. Staffing and Antipsychotic Medication Use in Nursing Homes and Neighborhood Deprivation. *JAMA Netw Open*. Published April 24, 2024.
doi:10.1001/jamanetworkopen.2024.8322

Data
Data available: Yes
Data types: Deidentified participant data
How to access data: Data will be made available upon reasonable request
When available: With publication

Supporting Documents
Document types: None

Additional Information
Who can access the data: Upon reasonable request
Types of analyses: for any purpose
Mechanisms of data availability: with reasonable request
Any additional restrictions: n/a
